# Connect Brain, a Mobile App for Studying Depth Perception in Angiography Visualization: Gamification Study

**DOI:** 10.2196/45828

**Published:** 2023-10-20

**Authors:** Andrey Titov, Simon Drouin, Marta Kersten-Oertel

**Affiliations:** 1 Software and Information Technology Engineering Department École de Technologie Supérieure Montreal, QC Canada; 2 Gina Cody School of Computer Science and Engineering Concordia University Montreal, QC Canada

**Keywords:** medical image visualization, volume visualization, depth cues, angiography, gamification, mobile games, mobile phone

## Abstract

**Background:**

One of the bottlenecks of visualization research is the lack of volunteers for studies that evaluate new methods and paradigms. The increased availability of web-based marketplaces, combined with the possibility of implementing volume rendering, a computationally expensive method, on mobile devices, has opened the door for using gamification in the context of medical image visualization studies.

**Objective:**

We aimed to describe a gamified study that we conducted with the goal of comparing several cerebrovascular visualization techniques and to evaluate whether gamification is a valid paradigm for conducting user studies in the domain of medical imaging.

**Methods:**

The study was implemented in the form of a mobile game, *Connect Brain*, which was developed and distributed on both Android (Google LLC) and iOS (Apple Inc) platforms. Connect Brain features 2 minigames: one asks the player to make decisions about the depth of different vessels, and the other asks the player to determine whether 2 vessels are connected.

**Results:**

The gamification paradigm, which allowed us to collect many data samples (5267 and 1810 for the depth comparison and vessel connectivity tasks, respectively) from many participants (N=111), yielded similar results regarding the effectiveness of visualization techniques to those of smaller in-laboratory studies.

**Conclusions:**

The results of our study suggest that the gamification paradigm not only is a viable alternative to traditional in-laboratory user studies but could also present some advantages.

## Introduction

### Background

In the field of medical imaging, angiography is used to visualize vascular structures inside the body. This is typically performed by injecting a contrast substance into a patient and imaging the patient via x-ray, magnetic resonance, or computed tomography [1]. For 3D x-ray, magnetic resonance, or computed tomography angiography (CTA), the result is a 3D volumetric representation of the scanned patient’s vascular anatomy. This 3D volume can be visualized using methods such as axis-aligned slicing [2], volume rendering, and surface rendering [3].

Cerebral angiography specifically depicts the blood vessels of the brain. The goal of this type of angiography is to help radiologists and surgeons understand the cerebral vasculature and detect abnormalities such as stenosis, arteriovenous malformations, and aneurisms [4]. However, visualizing angiography data such that they can be spatially well understood presents certain challenges [1,4,5]. First, the cerebral vasculature is complex, with intricate branching and many overlapping vessels, which hinders the understanding of the data in 3D [1,6]. Second, owing to variations in anatomy from patient to patient, surgeons may not always be able to rely on past experience to understand a new data set [1]. Third, depending on the environment (eg, the operating room), not all visualization methods might be suitable for rendering the data. For example, stereoscopic viewing requires specialized equipment (eg, a stereoscopic display or augmented reality glasses), which is not always available. Perspective rendering may also be inconvenient to use when displaying the data, as radiologists and surgeons may want to perform measurements on the angiographic image [4]; therefore, orthographic projection is most commonly used for 3D medical image visualization [1,4].

### Motivation

To improve the depth perception and spatial understanding of vascular volumes, numerous perceptually driven vessel visualization methods have been developed [3,4,7-10]. An overview of the most related studies and their results is presented in Table 1. The studies were chosen based on whether they contained algorithms that could be implemented with direct volume rendering (DVR). In addition, we focused exclusively on static visualizations, as in some contexts (such as the rendering of virtual vessels in augmented reality during a surgical intervention), it is not possible to have dynamic transformations. Thus, to limit the number of conditions and achieve more uniformity among the conditions, we focused only on static visualizations.

In all these works, user studies for determining the effectiveness of different visualization techniques were conducted in a laboratory environment under the supervision of a researcher [12]. This type of laboratory study has a number of disadvantages: the lack of diversity between the participants (who are often young college students) [12] and a limited pool of participants or, conversely, a high monetary cost for studies that have many participants [13]. As can be seen in the table, the number of participants per study was typically between 10 and 20. To overcome these issues, alternative user study paradigms such as crowdsourcing and gamification were explored [12].

Although crowdsourcing has previously been used to evaluate medical image visualization techniques [8,9], to the best of our knowledge, gamification has not been previously used for psychophysical experiments that study the effectiveness of medical visualization techniques. In our study, we used the gamification paradigm to collect data on the effectiveness of different perceptually driven vascular volume visualization techniques. Specifically, we developed a mobile app, *Connect Brain*, with 2 different games that we distributed on the web. The app was published on Google Play (Google LLC) [14] and the App Store (Apple Inc) [15]. Using the developed game, we evaluated the possibility of using the gamification paradigm to conduct user studies on medical imaging. Specifically, the developed game had similar research questions and metrics to those in prior laboratory studies (eg, the studies by Kersten-Oertel et al [1], Ropinski et al [4], and Abhari et al [6]) that evaluated the effectiveness of diverse cerebral vessel visualization techniques. We introduced specific gamification elements, such as levels, points, and leaderboards, to engage the participants and made the games available on the App Store [15] and Google Play [14] to reach a wider participant base. This paper is based on chapter 3 of the first author’s master’s thesis [16].

**Table 1:** Related works on depth volume rendering vascular visualization techniques.

Study	Visualizations	Participants, n	Trials and sample points	Goals	Metrics
Ropinski et al [4]	Phong, stereo, chroma, pseudochroma, overlaid edges, blended edges, perspective edges; edge shading; DoF^a^; and DoF+pseudochroma	14	50 × 14 = 700	Depth comparison	Correctness, time, and user feedback
Abhari et al [6]	No cue and edge	10	60 × 10 = 600	Connectivity	Correctness, time, and expert feedback
Kersten-Oertel et al [1]	No cue, kinetic, stereo, edge, pseudochroma, and fog+combined cues (for novice experiments only)	2 studies: 13 novices and 6 experts	160 × 13 = 2080 (novice); 6 × 50 = 300 (expert)	Depth comparison	Correctness, time, and user feedback
Drouin et al [7]	Shading, pseudochroma, fog, dynamic shading, dynamic pseudochroma, and dynamic fog	20	80 × 20 = 1600	Depth comparison and targeting or reaching	Correctness, time, pointer-target distance, and user feedback
Kreiser et al [10]	Phong, chroma, pseudochroma, VSS^b^ chroma, and VSS pseudochroma	19	150 × 19 = 2850	Depth comparison	Correctness and time
Titov et al [11]	Shading, pseudochroma, fog, dynamic shading, dynamic pseudochroma, and dynamic fog; all cues were visualized with a VR HMD^c^	12	80 × 12 = 960	Depth comparison and targeting or reaching	Correctness, time, pointer-target distance, head movement, and user feedback

^a^DoF: depth of field.

^b^VSS: void space surface.

^c^VR HMD: virtual reality head-mounted display.

### Gamification

Gamification is similar to crowdsourcing and shares its advantages [12]. Crowdsourcing is a method of conducting user studies that distributes a given task to a larger network of participants [12]. An example of a platform for crowdsourcing is the Amazon Mechanical Turk (MTurk) [17], which has been used in studies on a variety of topics, such as the perceptual effectiveness of line drawings to depict shapes [18], natural language processing [19], and audio transcription [20]. Crowdsourcing enables a larger study population than traditional methods because the task can be distributed on the web. In addition, the participant pool becomes more diverse because the study is no longer limited to a physical environment (eg, a university laboratory). Finally, crowdsourcing is less time consuming for each individual participant and allows a lower per-participant cost [17]. This model also has some disadvantages; the main disadvantage being low data quality because researchers do not have much control over the unfolding of the experiment and because participants may be motivated only by monetary gain [12,13].

The main difference between gamification and crowdsourcing is that gamification introduces gaming elements to the study [12]. Through gamification, a study is transformed into a game that is fun to play, and the gameplay data are collected and analyzed as the results of the study. The most important advantage of gamification is that users are motivated to perform well, which consequently increases the quality of the collected data compared with crowdsourcing. Further, players are motivated to perform well not because of monetary incentives but because they enjoy playing the game [13]. As gamification scales well with a large number of participants (because players download and play the games on their own devices), these types of studies have an even lower runtime cost than crowdsourcing [13]. However, there are several disadvantages. First, not every study can be transformed into a game that is fun to play. Furthermore, developing and publishing a game requires more time and effort than creating an experimental task. Finally, for success, the researcher should develop interesting game mechanics that follow the rules of game design [13].

The goal of our work is to determine whether the gamification paradigm is a valid approach to performing user studies, specifically in the context of medical imaging.

## Methods

### Overview

Connect Brain was developed using the Unity engine (Unity Technologies) [21] for the Android and iOS platforms. Before starting to play the game, all players had to provide informed consent for their gameplay data to be collected anonymously and used for research purposes. They could do this by manually checking the corresponding box during the initial profile creation. In addition, an email address was provided in case players had any questions regarding the user study.

A total of 7 different visualizations were implemented in the mobile app: Blinn-Phong shading [22], edge enhancement [23], aerial perspective (also called fog) [5], chromadepth [24], pseudochromadepth [4], and chromadepth and pseudochromadepth versions of void space surfaces (VSSs) [10]. In all visualizations, the medical data set was rendered using real-time DVR. Note that all visualizations are shaded using the Blinn-Phong shading model in addition to the specified method.

### Implemented Visualizations

**Figure 1:**
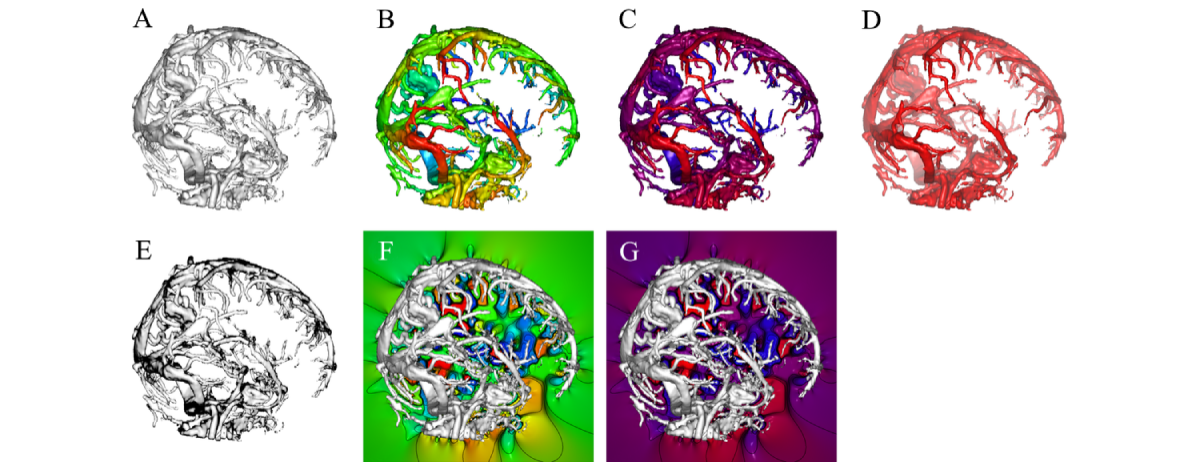
All the implemented vessel visualization techniques: (A) shading (Blinn-Phong), (B) chromadepth, (C) pseudochromadepth, (D) aerial perspective, (E) edge enhancement, (F) void space surface (VSS) chromadepth, and (G) VSS pseudochromadepth.

In the following section, we describe the details of the vascular volume visualization techniques (Figure 1) that were implemented in the Connect Brain game.

*Blinn-Phong shading* [22] is a photorealistic illumination model that describes how a surface reflects light when illuminated by one or multiple light sources. Similar to Drouin et al [7], we used it as the baseline visualization technique. In our implementation, a single-directional light source was used whose direction was parallel to the view direction (Figure 1A). In terms of color, both the volume and the light source were white.

*Edge enhancement* is used to emphasize the occlusion depth cue, where a viewer determines the relative depth between different objects based on the way they overlap [23]. In vessel visualization, the contours of vessels are emphasized, typically by rendering dark lines around the edges of the vessels [25] (Figure 1E). This cue is especially helpful when the transfer function (TF) produces a translucent result. In this case, the highly contrasted black silhouettes occlude the silhouettes of the vessels that are farther away from the viewer, thus providing a better understanding of the depth ordering of vessels.

Following the work of Drouin and Collins [23], in our implementation, edge enhancement was combined with Blinn-Phong shading. To do this, the volume is rendered using Blinn-Phong shading, and each pixel that forms the silhouette is darkened based on its interpolated normal vector. Pixels with a gradient that is almost perpendicular to the viewer are considered part of the silhouette. Drouin et al [23] described the following formula for calculating the intensity of edge enhancement for a given pixel:



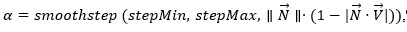



**(1)**

where *α* is the intensity of the edge enhancement factor, 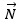 is the gradient (normal vector) of the surface, 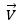 is the direction of the ray (from the volume toward the viewer), and *stepMin* and *stepMax* are user-defined parameters.

*Aerial perspective* (sometimes referred to as fog) is a monocular depth cue caused by the atmosphere and the way in which light scatters. Specifically, the farther the distance between an object and a viewer, the less contrast there is between the object and the background. With this technique, the vessels that are closer to the viewer appear more saturated and more contrasted, whereas farther vessels fade into the background [1,5] (Figure 1D). By comparing the saturation of 2 vessels, it is possible to deduce which one is closer and which one is farther away.

To render a data set with an aerial perspective cue, the pixels representing the color should be correctly blended with the background. Rheingans and Ebert [26] described the following formula for distance-color blending:

*C = (1 – d) c_o_ + d c_b_* **(2)**

where *d* is the depth of the volume at the current pixel in the range of {0,1}, *c_o_* is the color of the object, and *c_b_* is the color of the background. Preim et al [5] noted that the relationship between the depth of the projected vessel and saturation of the pixel does not need to be linear but can rather be exponential (by replacing *d* with an exponential function). To ensure the visualization of the entire volume (such that no vessels are blended completely into the background), Kersten et al [27] determined that the best upper bound for *d* was between 0.75 and 0.85. In our implementation, we used the original linear formulation with *d*=0.8.

*Chromadepth*, a technique developed by Steenblik [28], encodes depth using color. Specifically, the color of the pixels in depth follows the colors of the visible light spectrum, starting from red; progressing through orange, yellow, green, and cyan; and concluding with blue [24]. Thus, for a vascular volume, the closest vessels are red, the farthest vessels are blue, and vessels in between have a color that is linearly interpolated between these values (Figure 1B). Bailey and Clark [24] described the chromadepth TF as a 1D texture containing all colors (from red to blue), where *s* is defined as the sampling parameter. *D_1_* and *D_2_* are parameters defined by the viewer such that *D_1_≥0*, *D_2_≥1*, and *D_1_<D_2_*, and for any depth *d* where *d ε {0,1}*, TF is defined as follows:

if *d<D_1_*, then the color of the pixel is red, and if *d>D_2_*, then the color of the pixel is blue; otherwise, 
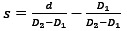
 **(3)**

**Figure 2:**
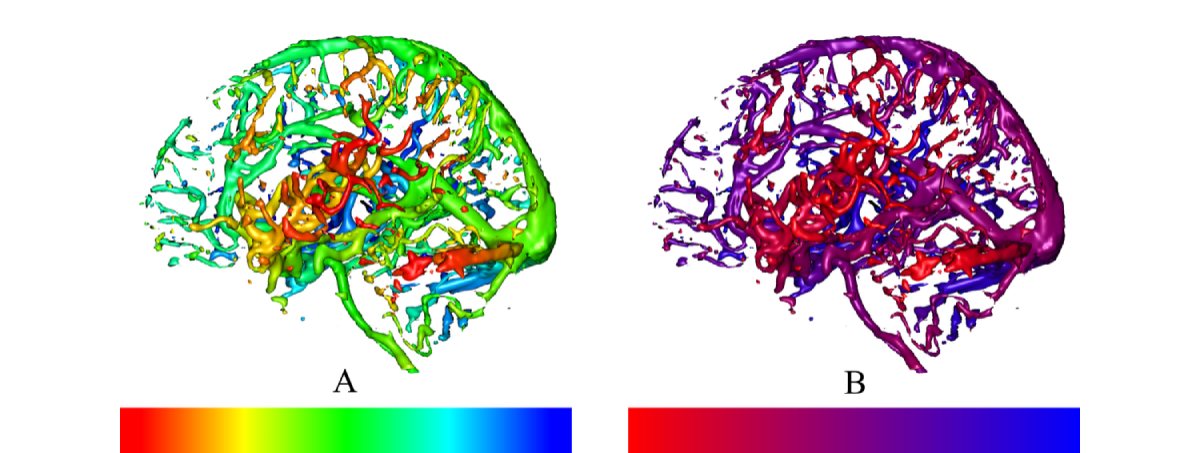
(A) Chromadepth and (B) pseudochromadepth with 1D transfer functions indicating near to far color mapping.

Figure 2A shows the TF used in our implementation for chromadepth as well as a sample volume shaded in this manner.

*Pseudochromadepth*, which incorporates only 2 colors (red and blue) instead of the full color spectrum, was used by Ropinski et al [4] to deal with the large number of hues presented in a chromadepth image, which can distract the viewer from the understanding of the depth. Red and blue colors are used (Figure 1C) because of the visual phenomena of chromostereopsis [29], which is caused by the light of different colors refracting into different parts of the retina in the eye depending on the wavelength. Chromostereopsis can be used to make red objects appear closer in depth than blue objects.

When using pseudochromadepth for vasculature, the closest vessels are red; the farthest vessels are blue; and for any intermediate depth, the color of the pixel is calculated by interpolating between red and blue. Thus, using the pseudochromadepth depth cue, a depth comparison between 2 shaded objects can be simplified to a simple comparison of the hue, with warmer hues representing closer objects and colder hues representing farther objects. The pseudochromadepth cue was implemented in the same way as chromadepth, with the only difference being that the 1D rainbow-like texture was replaced by one where the color is linearly interpolated between red and blue, as shown in Figure 2B.

*VSS*, a technique used in vessel visualization, was developed by Kreiser et al [10] (Figures 1F and 1G). Unlike many other vessel visualization techniques that are based on shading the vessels in a certain manner, VSS concentrates on shading the area around the vessels; the background is colored to indicate the relative depth of the surrounding vessels. Therefore, to understand the relative depth of a certain vessel, one must look at the color of the background that surrounds the vessel. The motivation behind VSS is that in more traditional depth rendering methods, there is a lot of unused empty space. Therefore, instead of being limited by the area that vessels occupy on the screen, the entire screen can be used, allowing the vessel pixels to represent any other information that may be deemed necessary.

To determine the color of each pixel, a weighted average of the depths of the surrounding border pixels is calculated. To do this, a rendered image of a vessel structure in the form of a depth map on which the filled pixels (representing the volume) can be distinguished from the empty pixels (representing the background) is required. The Suzuki and Abe [30] border-following algorithm is then executed on the depth map, creating a hierarchy of the borders of the depth map. This hierarchy indicates what border pixels contribute to what part of the background. Subsequently, the interpolated depth for each background pixel is calculated using inverse distance weighting [31]:



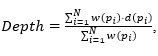



**(4)**

where *Depth* is the calculated depth of the background pixel, *p_i_* is the *i*th border pixel whose depth is used in the weighted average calculation, *N* is the total number of border pixels that affect the depth of *p_b_*, *w (p_i_)*, is the weight of the border pixel *p_i_*, and *d (p_i_)* is the depth of the border pixel *p_i_*.

The weight *w (p_i_)* of a border pixel *p_i_* is calculated in the following manner:



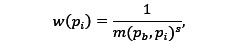



**(5)**

where *p_b_* is the background pixel for which the depth calculation is performed, *p_i_* is the *i*th border pixel whose depth is used in the weighted average calculation, *m (p_b_*, *p_i_)* is the magnitude of the vector between the position of the pixel *p_b_* and *p_i_*, and *s* is a user-defined smoothing parameter that results in closer border pixels giving exponentially more weight.

After calculating the depth of every background pixel, a TF is applied to the depths, transforming them into a color. Typically, chromadepth (Figure 1F) and pseudochromadepth (Figure 1G) are used [10]. In addition, VSS implements an approximated version of global illumination in the form of screen space directional occlusion (SSDO) [32]. SSDO darkens some regions of the generated VSS that may be occluded from the light emitted by neighboring parts of the VSS and performs an indirect light bounce. Finally, isolines are generated on the surface of the VSS in the form of black lines to improve the understanding of the generated shape by the VSS.

Owing to the hardware limitations of mobile devices, we used screen space ambient occlusion [33] instead of SSDO, which does not include indirect bounce.

### Ethics Approval

The user study was approved by the Natural Sciences and Engineering Research Council of Concordia University (certification 30016074).

### DVR on the Mobile Device

To visualize the volumes, the DVR technique described by Drouin et al [23], which is based on a well-known 2-pass rendering algorithm described by Kruger et al [34], was used. This technique describes a real-time ray casting algorithm that consists of 2 rendering passes. In the first pass, the front and back faces of a colored cube representing the bounding box of the volume are rendered into 2 different textures. The red, green, and blue colors encode the start and end positions (as 3D coordinates) of the ray for each pixel. In the second pass, for each pixel, a ray is sent through the volume, and the opacity is accumulated while sampling the volume using trilinear interpolation. The ray stops, and the distance traveled by the ray is recorded into a third texture. Next, a compute shader scans the third texture to determine the smallest and highest nonzero depths of the texture such that the visible interval of the volume inside the 3D texture is known. Finally, in the second pass, the final image of the volume is rendered using the recorded pixel depths, which are adjusted using the minimum and maximum values calculated previously so that the entire range of depth values (from 0 to 1) lies within the visible part of the volume. A TF maps the adjusted depth values to the red, green, blue, and alpha colors for each pixel. This TF is encoded as a 1D texture that is passed to the shader.

As mobile device graphics processing units are typically slower than their desktop equivalents, additional optimizations were made to allow for real-time rendering. First, the ray casting algorithm was simplified so that instead of accumulating opacity at each ray step until full opacity was reached, the ray stopped immediately when the sampled value in the volume reached a given threshold, similar to the early ray termination described by Levoy [35]. Second, the ray casting algorithm was modified to reduce the frequency at which the volume was sampled. To achieve this, the 3D Chamfer distance approach described by Zuiderveld et al [36] was used. This method speeds up ray casting without compromising the quality of the rendered image by determining the distance to the closest nonzero voxel for every voxel and storing it in a 3D texture. This distance corresponds to the number of voxels that must be traversed to create a path in 3D space, assuming a 26-cell cubic neighborhood. Here, a small threshold value was defined to distinguish the *empty* voxels from the nonempty voxels. When performing ray casting, the value from the Chamfer distance 3D texture, which indicates the distance that the ray can safely travel without missing any interesting voxels, is used. Thus, the empty areas of the volume are traversed faster. It should be noted that although the algorithm does not compromise the quality of the volume, it requires more space to store the additional volume.

Finally, to save the battery life of the mobile device and have a smoother user interface, when the volume is not being rotated, it is rendered once to a texture and then displayed in future frames. In addition, when the volume is rotated, it is temporarily downscaled during ray casting, the smaller volume is rendered to a temporary frame buffer, and then the image obtained from this frame buffer is upscaled using linear interpolation. The intensity of the downscaling is directly proportional to the speed of the rotation of the volume, making the downsampling less perceptible to the viewer.

Using these optimizations, real-time rendering was achieved on the mobile devices tested for all cues except VSSs. Despite attempts to improve the calculation time of VSS, rendering times of only a few seconds per frame were achieved. As a result, a static version of VSS that cannot be interacted with was used in Connect Brain.

### Connect Brain Gameplay

**Figure 3:**
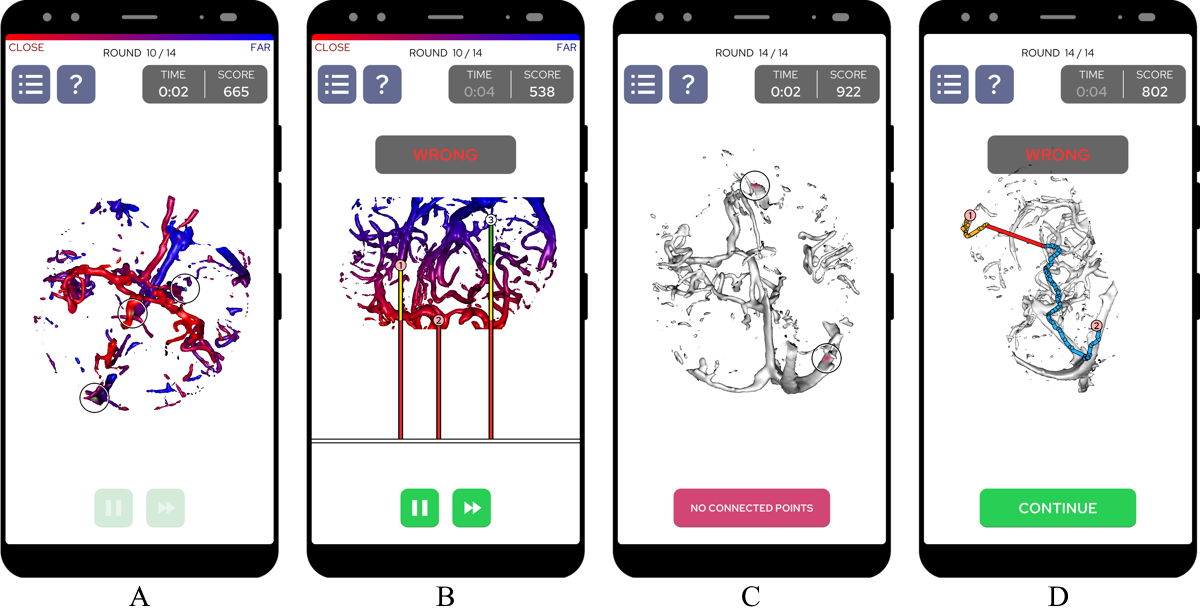
Connect Brain screenshots: (A) gameplay of the Near-Far Game, (B) feedback for the Near-Far Game, (C) gameplay of the Blood Circulation Game, and (D) feedback for the Blood Circulation Game. Phone frame source: adapted from Wikimedia. The original uploader was MDXDave at German Wikipedia, CC BY-SA 3.0.

Connect Brain consists of two minigames: (1) the *Near-Far Game*, a game in which players compare the relative depth between the indicated vessels, and (2) the *Blood Circulation Game*, a game in which players must understand the connectivity between different points in the vascular volume (Figure 3, where the phone frame was adapted from Wikimedia [37]; the original uploader of the frame was MDXDave at German Wikipedia, CC BY-SA 3.0 [38]). Both minigames are split into a tutorial level that teaches the player the basics of the minigame and 11 levels that can be played in any order after the completion of the tutorial. Each level is defined by 4 parameters: the CTA data set used, threshold used for early ray termination, depth of the near and far clipping planes, and number of points selected on the volume (≥2). Each level in the game consists of 14 rounds in total, with each round showing a single visualization among those that were implemented. A legend was always present to help the players understand the color encodings for each visualization, and the player could also read the description of the visualization by pressing on a question mark icon. To avoid confusing the player and prevent biases, we decided to use the same visualization technique for 2 consecutive rounds before randomly selecting a new visualization. Videos demonstrating the gameplay of these games can be found in the multimedia appendices (see Multimedia Appendix 1 for the Near-Far Game and Multimedia Appendix 2 for the Blood Circulation Game).

### Near-Far Game

The *Near-Far Game* focuses on understanding the relative depth between vessels. This game is based on the experimental task described and used by Ropinski et al [4], Kersten-Oertel et al [1], and Kreiser et al [10]. The typical experimental task involves participants determining the nearest vessel between 2 selected vessels rendered using a given visualization technique. The Near-Far Game in our app uses the same principle but introduces some gameplay elements to make it more fun for players.

Players are presented with a CTA on which ≥2 points on vessels are indicated. The task of the player is to connect the points from the point closest to them to the point farthest from them using their finger. The points are indicated on the volume using a contrasting color, and to ensure that they are visible, a black and white circle is placed around them (Figure 3A). This circle also indicates the region where the player can touch the screen to select the point. To further help indicate the positions of the points, arrows appear on the screen, indicating the location of the points during the first second of each round. The selected points and view of the CTA are randomly chosen, meaning that the player cannot simply learn the correct answers. This also makes replaying a level more interesting, as the player will always have new data to view and interact with. Although random, a number of rules are applied to choose the points: (1) they are always clearly visible from the player’s perspective; (2) they have a small minimum depth difference between them; and (3) there is a minimum *xy* pixel position difference between them, which is equal to the diameter of the black-and-white circle × 1.5 to avoid the overlapping of 2 indicator circles.

By connecting the points in the correct order, the player gains score points; and additional bonus points are provided for doing this quickly. The number of bonus points is calculated by applying a reciprocal function to the round time. However, if the player makes an incorrect decision, the bonus is subtracted from their current score. This gives players an incentive to complete rounds as fast as possible while simultaneously motivating them to make accurate decisions. Further, the score accumulates through the rounds and is saved on a global leaderboard where players can compare their score to others. The score of a player is only visible to other players if it is one of the top 3 scores for the current level, and this setting cannot be changed.

Some levels have rounds in which >2 points are indicated to the player. In these rounds, the player can connect any number of points at once. The goal in this case is to select all the connected points in the ascending depth order, starting from the closest point in terms of depth (similar to the work of Ritter et al [3]). However, if a point with a larger depth is selected before a point with a smaller depth, then the entire selection is considered incorrect, and the player loses the bonus time points. If all the points are connected in the correct order, the player will receive significantly more points than if they connected each pair of points individually. Thus, selecting multiple points at once is a high-risk, high-reward strategy.

During gameplay, we enable players to rotate the volume as a last resort measure when they get stuck at a certain round. The players can rotate the volume with an offset of up to 45° from the initial position. If *x* and *y* are the rotation in degrees around the *x-* and *y-*axis from the initial position, then the rotation of the volume always follows the formula 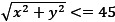 . To discourage rotation (as we wanted players to understand the data using the given visualization technique), we designed the game such that players lose score points for rotating the volume. The amount of points lost is directly proportional to the rotation of the volume in degrees. This feature was added to reduce the frustration of the player and lower the chance that they will completely abandon the game.

A preliminary in-laboratory study was conducted with 12 participants to test the gameplay aspect of Connect Brain. One of the findings of this preliminary study was that users wanted to know how they were wrong when they made an incorrect decision. Thus, a feedback feature was added; if enabled, at the end of each incorrectly completed round, the volume is rotated by 90° around the x-axis so that the points that are closer to the viewer are positioned on the bottom of this view and the points that are farther are positioned on the top. Vertical lines are then drawn like a ruler to demonstrate the relative depth between the points (Figure 3B).

### Blood Circulation Game

The *Blood Circulation Game* focuses on the connectivity between different vessels in the vascular volume. This game is an adaptation of the experiment that was described by Abhari et al [6], in which participants were presented with static 2D images and asked to determine whether a path exists between 2 selected points on the visible vessel structure. We built on this experiment by adding motivating gameplay features to it.

As in the *Near-Far Game*, players are presented with ≥2 points selected on the vascular volume. However, the goal of this game is to determine which points are directly connected, in other words, whether a path exists between the 2 vessels. As each selected point on the 2D image is associated with a specific voxel in 3D, connectivity refers to the path between the 2 voxels inside the 3D volume. When the player finds 2 connected points, they link them using their finger in any order. However, if no 2 points seem to be connected with each other, the player should press the “no connected points” button that is located at the bottom of the screen (Figure 3C).

As described in the first game, the initial rotation of the volume at the beginning of each round and the selection of points are performed randomly. This means that we need to compute at runtime whether 2 voxels are connected with each other within the 3D data set. To achieve this, the A* search algorithm [39], which determines the path (if it exists) between 2 voxels inside a 3D texture, was used. A* is an informed search algorithm that considers both the distance traversed so far and an estimation (heuristic) of the remaining path, allowing it to perform very quickly and find the optimal path in case the heuristic function is admissible (never overestimates the cost to reach the goal). This algorithm requires a priority queue data structure to function, and we chose the Fibonacci heap [40] because of its efficient performance. The threshold used to define the boundaries of the vessels during path finding is the same as that used for ray casting.

The score system works in the same manner as in *Near-Far Game*, with points awarded for correct decisions about whether a path exists and for fast decision response times. The rotation of the volume also works in the same manner, resulting in a loss of points.

The Blood Circulation Game also features a feedback system; if the player decides that 2 points are connected, but in fact they are not, the feedback view shows the minimum distance that separates the 2 independent parts of the vessel structure. Conversely, if the player decides that no points are connected with each other, but some of them are, then this view demonstrates the path between the connected points (Figure 3D).

Once Connect Brain was made available on the Apple App Store and Google Play, we advertised it not only on various social media channels, such as LinkedIn [41], Twitter [42], and Facebook [43], but also through email lists to encourage users to play.

## Results

### Overview

At the time of our analysis, a total of 111 participants (men: n=68, 61.3%; women: n=39, 35.1%; nonbinary: n=4, 3.6%) had downloaded and played the mobile game. In addition to the 111 participants who played the game, 21 others downloaded it but did not play. Of the 111 participants, 54 (48.6%) played on Android, and the remainder (n=57, 51.4%) played on iOS. Owing to the restriction on the collection of age data on iOS apps, age was collected only from the participants who used the Android version; the age range of these participants was from 14 to 62 (mean 30, SD 11) years. Among the 111 participants, 50 (45%) had experience with medical visualization, 30 (27%) were familiar with angiography, and 36 (32.4%) had experience with vessel visualization techniques. More precisely, of the 111 participants, 26 (23.4%) had experience in all 3 previously listed domains (we refer to them as experts), and 31 (27.9%) had experience in either 1 or 2 domains (we refer to them as semiexperts). All 111 (100%) users participated in the Near-Far Game, completing, on average, 39 (SD 61) rounds, but only 44 (39. 6%) players participated in the Blood Circulation Game, completing, on average, 37 (SD 39) rounds. We hypothesize that the reason why some participants decided to quit the game too early was because they were playing the game in an environment that was not controlled, so they could stop at any moment if they were bored or did not want to continue playing. It is also possible that some players downloaded the game without knowing its purpose and were simply uninterested in playing after downloading. An ANOVA and a post hoc Tukey honest significant difference tests were used to measure and analyze correctness and response time variables. This analysis was performed using the SPSS software (version 26; IBM Corp) [44].

Similar to Kersten-Oertel et al [1] and Lawonn et al [9], for both games, in addition to correctness and response time, we examined the effect of both the distance between the indicated vessels on the screen (*xy* distance) and the distance in depth between the indicated vessels (*z* distance). Both *xy* and *z* distances were equally divided into 2 categories, *near* or *far*, measured in world coordinates. For the *xy* variable, the ranges are defined in the following manner: near (0.162-0.369) and far (0.369-0.951). For the *z* variable, the ranges are defined as follows: near (0.021-0.104) and far (0.104-0.792; note that *z* distances are distributed unequally because the close and far clipping planes in some levels greatly limit the total depth range of the volume, resulting in a larger number of entries with a small depth distance).

Owing to a lack of control over the timing and how the game was played (eg, a person might get interrupted during the game, thus increasing the decision time), we removed all extreme outliers equal to *Q_3_ + 3* × *IQR*, where *Q_3_* represents the value at the third quartile and IQR equal to *Q_3_ – Q_1_*. In addition, we discarded all data completed during the tutorial levels.

### Near-Far Game

A total of 5367 entries were collected for the Near-Far Game. In cases where multiple points (3 or 4) were connected simultaneously, each individual pair of connected points was treated as an individual entry.

#### Correctness

Correctness was represented by either 1 (correct) or 0 (incorrect) and determined based on whether the connection between points was done in the correct order. The mean correctness and SE for each visualization method are shown in Table 2. A 3-way repeated measures ANOVA was used to examine the main effects as well as the interactions of the visualization method, *xy* distance, and *z* distance, as they relate to correctness. The ANOVA showed that the visualization method had a significant effect on correctness (*F*_6,5339_=22.404; *P*<.001). A Tukey post hoc test showed that pseudochromadepth (mean 83%, SE 1.5%), aerial perspective (mean 82%, SE 1.5%), and chromadepth (mean 81%, SD 1.5%) allowed for better depth perception than VSS chromadepth (mean 72%, SE 1.6%), VSS pseudochromadepth (mean 72%, SE 1.6%), edge enhancement (mean 66%, SE 1.6%), and shading (mean 65%, SE 1.6%). Although both VSS versions performed better than shading and edge enhancement, only the difference with shading was found to be statistically substantial according to the Tukey honestly significant difference test.

We found a significant main effect of distance on correctness (*F*_1,5339_=24.708; *P*<.001). As expected, the near *z* distance (mean 71%, SE 0.9%) resulted in worse correctness compared with the far *z* distance (mean 77%, SE 0.8%). However, we found no main effect of the *xy* distance on correctness (*F*_1,5339_=1.329; *P*=.25). Moreover, there was no significant 2-way interaction between *xy* distance and visualization method on correctness of depth ordering (*F*_6,5339_=0.627; *P*=.71), between *z* distance and visualization (*F*_6,5339_=1.836; *P*=.09), or between the *xy* and *z* distances (*F*_1,5339_=0.619; *P*=.43). There was also no significant 3-way interaction between the variables (*F*_6,5339_=0.595; *P*=.74).

**Table 2:** Mean correctness and decision time for the Near-Far Game, depending on the visualization that was used^a^.

	Correctness (%), mean (SE)	Time (s), mean (SE)
Arial perspective	82 (1.5)	4.77 (0.117)
Shading	65 (1.6)	5.29 (0.120)
Chroma	81 (1.5)	5.03 (0.117)
Edges	66 (1.6)	4.98 (0.12)
Pseudochroma	83 (1.5)	4.89 (0.118)
VSS^b^ chroma	72 (1.6)	5.58 (0.122)
VSS pseudochroma	72 (1.6)	5.44 (0.122)

^a^Error bars represent the SE.

^b^VSS: void space surface.

#### Decision Time

The decision time for levels with 2 points corresponds to the interval between the moment when the round starts, *T_o_*, and the moment when the finger of the player reaches the second point, *T_2_*. When >2 indicated vessels (ie, *n*) are connected in the same level, the time for connecting *n– 1* with *n* is calculated as *T_n_ = T_1_ + T_n_ – T_n–1_*. Thus, we consider the time taken to touch the first indicated vessel, which we consider the time taken by the player to make decisions about the spatial layout of the vasculature as a whole, plus the time interval to connect the 2 indicated vessels *n – 1* and *n*. The mean decision time and SE for each visualization method is shown in Table 2.

A 3-way repeated measures ANOVA was used to examine the main effects and interactions of visualization methods, *xy* distance, and *z* distance on decision time. The ANOVA showed that the visualization method had a significant effect on response time (*F*_6,5339_=6.334; *P*<.001). A post hoc Tukey test showed that aerial perspective (mean 4.77, SD 0.117 s) and pseudochromadepth (mean 4.89, SE 0.12 s) resulted in the fastest decision times and performed better than both VSS chromadepth (mean 5.58, SE 0.12 s) and VSS pseudochromadepth (mean 5.44, SE 0.12 s). However, only aerial perspective performed better than shading (mean 5.29, SE 0.12 s), which had the third worst decision time. Chromadepth (mean 5.03, SE 0.12 s) and edge enhancement (mean 4.98, SE 0.12 s) were faster than VSS chromadepth but not VSS pseudochromadepth.

There was a significant main effect of *xy* distance (*F*_1,5339_=12.630; *P*<.001) on decision time. Far *xy* distances (mean 5.30, SE 0.06 s) resulted in longer decision times than near *xy* distances (mean 4.98, SE 0.06 s). In addition, there was a significant main effect of *z* distance (*F*_1,5339_=12.924; *P*<.001) on decision time. Far *z* distances (mean 4.98, SE 0.06 s) resulted in a shorter decision time than near *z* distances (mean 5.30, SE 0.07 s).

There was no significant 2-way interaction between the visualization method and the *xy* distance (*F*_6,5339_=0.476; *P*=.83), the visualization method and the *z* distance (*F*_6,5339_=1.190; *P*=.31), or the *xy* distance and the *z* distance (*F*_1,5339_=0.063; *P*=.80). There was no 3-way interaction either (*F*_6,5339_=1.455; *P*=.19).

### Blood Circulation Game

The total number of entries collected for the Blood Circulation Game was 1810. A 3-way repeated measures ANOVA was used to examine the main effects as well as the interactions of visualization method, *xy*-distance, and *z*-distance, as they relate to correctness and response time for the Blood Circulation Game.

#### Correctness

Correctness in the Blood Circulation Game corresponds to whether the player correctly identified the indicated vessels as connected (Table 3). The ANOVA showed that there was no main effect of visualization technique (*F*_6,1782_=1.383; *P*=.22), *xy* distance (*F*_1,1782_=0.032; *P*=.86), or *z* distance (*F*_1,1782_=0.004; *P*=.95) on correctness. Furthermore, there was no significant 2-way interaction between the visualization method and *xy* distance (*F*_6,1782_=0.867; *P*=.52), between the visualization method and *z* distance (*F*_6,1782_=1.406; *P*=.35), or between *xy* distance and *z* distance (*F*_1,1782_=2.251; *P*=.13). No significant 3-way interaction was found either (*F*_6,1782_=1.536; *P*=.16).

**Table 3:** Mean correctness and decision time for the Blood Circulation Game, depending on the visualization that was used^a^.

	Correctness (%), mean (SE)	Time (s), mean (SE)
Arial perspective	80 (2.4)	3.46 (0.135)
Shading	80 (2.5)	3.18 (0.137)
Chroma	81 (2.5)	3.4 (0.138)
Edges	84 (2.4)	3.27 (0.136)
Pseudochroma	87 (2.4)	3.11 (0.133)
VSS^b^ chroma	81 (2.5)	3.52 (0.138)
VSS pseudochroma	80 (2.5)	3.49 (0.141)

^a^Error bars represent the SE.

^b^VSS: void space surface.

#### Decision Time

The mean decision time and SE for each visualization method are shown in Table 3. ANOVA showed that there was a significant 2-way interaction between the *xy* and *z* distances on correctness (*F*_1,1782_=4.583; *P*=.03). The combination of far *xy* and far *z* distances correspondingly resulted in a substantially longer decision time (mean 3.59, SE 0.11 s) than any other combination. There were no significant main effects of visualization method (*F*_6,1782_=1.441; *P*=.20), *xy* distance (*F*_1,1782_=1.550; *P*=.21), or *z* distance (*F*_1,1782_=1.559; *P*=.21) on decision time. No significant 2-way interactions were found for the visualization technique and the *xy* distance (*F*_6,1782_=1.409; *P*=.21) or for the visualization technique and the *z* distance (*F*_6,1782_=1.044; *P*=.40). Finally, no 3-way interaction was found either (*F*_6,1782_=0.708; *P*=.64).

## Discussion

In general, we found that our results match those of studies that contain a larger number of participants, which suggests that the gamification paradigm is a viable alternative to conducting studies in the domain of medical imaging and, more precisely, angiography visualization.

### Depth Perception and Connectivity

The analysis of the gameplay data showed that aerial perspective, chromadepth, and pseudochromadepth allow for the best relative depth perception. These techniques led to the most correct responses and the quickest times, although only aerial perspective resulted in a faster decision time than shading. For vessel connectivity, no cue performed substantially better than the others.

Similar to the study by Kersten-Oertel et al [1], we found that for depth perception, the aerial perspective and pseudochromadepth visualization techniques performed very well in terms of both correctness and decision time. However, unlike Kersten-Oertel et al [1] and Ropinski et al [4], who found pseudochromadepth to be significantly better than chromadepth, we found no difference between the cues. However, this is in line with the results reported by Kreiser et al [10], who found no difference between these 2 cues.

As for the VSS cues, we found that they performed slightly worse compared with the results obtained by Kreiser et al [10]. Although VSS chromadepth and VSS pseudochromadepth resulted in a substantially higher accuracy than shading, both performed worse than the non-VSS versions of chromadepth and pseudochromadepth. In terms of decision response time, we found a similar result to that found by Kreiser et al [10]; VSS had longer times than the directly applied visualization methods. This can be expected owing to the indirect nature of this vessel visualization technique. The correctness results may be explained by the fact that the visualized vasculature is complex, and on small devices (eg, smartphones), there is a limited amount of background, which is needed for VSS. In addition, because of the hardware limitations of mobile devices, VSS was the only cue that was not adjusted in real time when the player was rotating the volume. However, despite this constraint, VSS cues still managed to be more effective than shading, so VSS would be preferable in a context where the color of the vessels cannot be changed.

Edge enhancement was not found to be an effective cue. In terms of depth perception, it resulted in the lowest correct responses, similar to shading. In terms of decision response times, it was substantially better than only VSS chromadepth, and VSS techniques are known to require a significant amount of time to understand. In terms of vessel connectivity understanding, unlike Abhari et al [6], edge enhancement did not improve accuracy or decision time. In fact, this visualization technique had no significant impact on either correctness or response time in terms of understanding vessel connectivity. We posit that this is the case because we tended to demonstrate simpler vessel structures in the Blood Circulation Game, which was achieved by using closer clipping planes to avoid having all vessels connected with each other. The negative side effect of this was that accuracy was high across all visualizations, and decision times were generally similar. These similarities in time could be explained by the fact that players rotated the volume using their finger, but even after removing all entries where players rotated the volume, no effect was observed on the decision time.

In terms of distances between the indicated vessels, as expected, having a far *z* distance between the vessels improves relative depth perception and, surprisingly, decision time, which is different from what was observed by Kersten-Oertel et al [1]. The reason behind shorter decision times at long *z* distances could be that with shorter z distances, the players had to resort to rotating the volume with their finger to understand the depth using motion parallax. Regarding *xy* distance, although it had no effect on accuracy, it did have an effect on the decision response times, with longer *xy* distances resulting in a longer decision time. This may have been caused by the fact that for longer *xy* distances, players had to perform a longer gesture when connecting the indicated vessels. By contrast, in the Blood Circulation Game, where players had to perform a similar gesture, a long *xy* distance resulted in longer decision times only when it was combined with a long *z*-distance, which could mean that the hand gesture does not have a big impact on the decision time. Another reason for this is that players may look back and forth between indicated vessels more often in case of longer distances.

For the Blood Circulation Game, the combination of long *xy* and long *z* distances resulted in the longest decision times. This may have been because in such a combination, the vessels were the farthest apart from each other, so players had to analyze the data set more carefully to draw any conclusion about the connectivity.

### Crowdsourcing and Gamification

In this paper, we describe the results of a study that compared the effectiveness of cerebral blood vessel visualization techniques, which was conducted using a mobile game, rather than in a traditional laboratory setting. Similar to previous studies, we found that aerial perspective, chromadepth, and pseudochromadepth allow for the best relative depth perception. In terms of determining the connectivity between 2 vessels, we found that the visualization method did not affect the result.

What differentiates our study from related works is the gamification paradigm that was used to conduct the study. Rather than having participants perform an experiment in a laboratory, we created a mobile game that was distributed using mobile app distribution platforms. Gamification presented multiple advantages compared with traditional in-laboratory user studies. First, it allowed us to have a high number of participants (111 at the time of analysis) with no additional per-participant cost. Second, the participants were also highly diverse, with 39 (35.1%) out of 111 participants identifying as women and 4 (3.6%) identifying as nonbinary. Third, gamification made it easier for us to recruit experts, as 16 (62%) out of 26 experts downloaded the app either from another country or another province of Canada, whereas among the semiexperts, this proportion was 18 (58%) out of 31. Finally, in cases where the study targets a broader range of participants, including nonexperts, gamification incentivizes the nonexperts to join because they might be interested in the game elements rather than the domain of the study. If we look at the average number of rounds completed by experts and semiexperts combined (mean 63, SD 107), it is approximately the same as that for nonexperts (mean 60, SD 83), which indicates that the interests of the 2 groups were approximately the same toward the game. We hypothesize that experts and semiexperts were primarily interested in continuing to play the game because of the domain of study, whereas nonexperts were interested because of the game elements, such as competing for a high score.

### Limitations

Gamification also presented some important disadvantages, both during the development of the game and with data collection.

First, transforming the experiment into a game that is fun to play required more development time and additional research to create interesting game mechanics. In our case, the user study could be transformed into a game because it integrated simple visual tasks for both minigames, which were visual comparison (in the Near-Far Game) and path finding (in the Blood Circulation Game). These tasks can both be used for an experiment, but they are also common game principles. However, by themselves, these visual tasks were not interesting enough to make the game fun, so additional game elements had to be added, such as the score system or high-risk, high-reward multiple connection mechanic.

Second, implementing volume rendering such that it allows real-time rendering on mobile devices required additional optimizations of the rendering code. In addition, to ensure that the game worked on different devices and operating systems, graphics processing units, resolutions, and aspect ratios also required additional development. Even though we tested our game on a variety of Android and iOS devices, we still could not guarantee that our game worked perfectly on all hardware configurations, as we received feedback from 1 (0.9%) of the 111 participants that one of the rendering techniques crashed on their device. In addition, we did not have control over the resolution or aspect ratio of the screen, which might have had an impact on performance. However, to achieve at least some consistency, we scaled the volume such that it was proportional to the vertical resolution of the screen.

Third, the lack of a controlled environment may have impacted the collected data. As we could not observe how the game was played, we cannot be sure whether players were motivated to try to do their best. At the same time, we think that adding a competitive element to the study in the form of a leaderboard did indeed motivate most players to perform well, which should have resulted in a higher quality of the collected samples. We also had little control over the credibility of the data that users filled when creating their account and could not create a detailed pretest or posttest questionnaire, which was not possible on iOS owing to privacy concerns and in general could lead to a player abandoning the game before even starting to play.

### Conclusions

Despite some of the drawbacks of gamification, using this paradigm allowed this study to collect more data samples than many similar studies [1,4,6,7,10]. Furthermore, it showed that our results were more similar to those of studies with more data samples and participants (2380 for Kersten-Oertel et al [1] and 2850 for Kreiser et al [10]) than those of studies with fewer samples (700 for Ropinski et al [4] and 600 for Abhari et al [6]). These results suggest that gamification is a viable paradigm for conducting user studies in the domain of medical imaging. Moreover, as demonstrated by our number of participants and results, if the game is fun to play and motivates the players to perform well in the study, it may lead to a higher number of participants compared with an in-laboratory user study while still maintaining a high quality of the collected data. Another advantage of web distribution-based paradigms, such as gamification, is that they make it possible to perform user studies or help with surgical education in societal situations where meeting in person is not possible [45]. Such was the case in this study, which was performed during the lockdown caused by the COVID-19 pandemic. Gamification is a promising technique for collecting large data samples; however, it is important to have fun games that users will continue to play. In the future, we could further improve the game by adding sound and music and examine whether these aspects have a positive impact on the time players spend in the game. In addition, we could pay the participants to play our game to determine how having a monetary incentive affects the behavior of the players, as they may enjoy the game more this way [46]. Regarding the study itself, in the future, illustrative techniques could be added to compare an even higher number of visualizations. Some good candidates are the hatching and distance-encoded shadows technique described by Ritter et al [3]; illustrative shadows, supporting lines, and contours technique described by Lawonn et al [9]; and anchors technique described by Lawonn et al [8]. Finally, we could compare our gamified user study to crowdsourcing, such as the EvalViz [47] wizard.

## Appendix

Multimedia Appendix 1 Gameplay video of the Near-Far Game.

Multimedia Appendix 2 Gameplay video of the Blood Circulation Game.

## Abbreviations

CTA: computed tomography angiography
DVR: direct volume rendering
MTurk: Amazon Mechanical Turk
SSDO: screen space directional occlusion
TF: transfer function
VSS: void space surface

## Data Availability

All the data used in the user study are available upon request from the corresponding author.
